# Observation on Mr. Morrison's Case of Ophthalmia

**Published:** 1808-09

**Authors:** 


### *To the Editors of the Medical and Physical Journal.*

GENTLEMEN,

WHEN the interests of society and humanity, when the happiness of numerous individuals, and when the cause of truth are at stake, silence would not only ill become, but would even be criminal in any person who feels, or professed, the least degree of affection for his fellow-creatures. Should such a being exist; should he console himself with the selfish idea, that, though the welfare of a world be involved in the question, yet as neither he nor his connections are liable to suffer by it, therefore there is not any necessity for his pleading the cause of veracity; I envy him not his feelings, though he rank ever so high in the scale of intellectual excellence.

Far be it from me, by what I have said above, to reflect upon any one. I hope the gentleman, upon whose communication I propose offering a few remarks, will be the last person to imagine such a thing. I am too well aware of the *delicacy* which *ought to be* observed in animadverting upon the writings of another; and, sorry am I to see, the spirit of *illiberal censure*, often approaching to *indecent scurility*, so widely disseminated among the Contributors to the Medical and Physical Journal. A candid and scientific examination into the opinions of others; an impartial and minute scrutiny of every discovery in medicine or surgery, that is brought before the public, would be of mutual advantage to the disciples of Esculapius, and to those who are destined to derive the benefits resulting from its excellence,—the human race.

In your Journal for July last, No. 113, is contained a case of the purulent ophthalmia, frequently occurring in young children soon after birth, by a Mr. Morrison of Dublin. At the conclusion of the case, are added some observations by the same gentleman, tending to prove that the above disease is produced by the *matter of lues venerea*, applied to the eyes of the child, during its passage through the vagina of the mother. To be decidedly of "opinion" of the proximate cause of a disease, judging merely from a single ambiguous case, is in every point of view certainly erroneous; and, more particularly in one so widely extended as the purulent ophthalmia; when we take into consideration, that the happiness of very many individuals and families may be irretrievably ruined by the assertions of of Mr. Morrison. From his account, it appears the child was affected with very severe symptoms of the disease, two days subsequent to its birth; and these symptoms originated from the application of venereal matter, produced in the vagina of the mother, to the tarsi of the infant. These circumstances are all doubtless correct, with the exception of the matter applied being *venereal*; and Mr. M's attempts to establish this point are extremely vague and unsatisfactory. In the first place, we are informed of nothing more, than that Mrs. N. had a venereal complaint, which had been suspended during the advanced period of utero-gestation. What was this complaint? and what were the symptoms attending it? Had she simple gonorrhœa, or had she lues, evinced by the presence of chancres, or diseased glands? For I coincide in opinion with most of modern surgeons, that the two diseases are perfectly distinct. Why was it "unequivocally", a case of the venereal kind? Did it require to be extirpated from the system by a mercurial course? These questions must be answered before an inquisitive mind can believe the affection was what Mr. Morrison asserts it to have been.

Respecting the method of cure adopted by Mr. M. to relieve the child's complaint, it is nothing more than what would have been eligible in an inflammatory affection of the eyes, proceeding from any other irritating cause; viz. the use of an astringent lotion, and the exhibition of calomel; though Mr. M, reasoning from the similiarity which existed in appearance between the ophthalmia and gonorrhœa, thinks the medicines acted in a specific manner. He promises to mention a "few other facts", in his "*observations*," to support his opinion; but on examination these dwindle down to one, namely, the experiment made with the matter discharged from the eyes of the infant, as applied to a small wound on the hand of a young man, and this is perfectly inconclusive. I neither wish, nor do I intend to call in question Mr. M's discriminative powers; yet, the single circumstance of the ulcer, produced by this application, taking on the "*appearance*" of the *venereal* sore, is not nearly sufficient to convince the least sceptical reader that it was so, when unaccompanied by any affection of the system, which, from Mr. M's silence on that head, I should imagine was the case. We know that acrimonious matter of an infinite variety of species, when applied to the surface of a wound, will produce a deranged secretion of matter from the mouths of the secerning arteries; and I am convinced this was the case in the above instance; instance; and with respect to the healing of the ulcer, during the taking of mercury, this is no proof: foul ulcers are frequently very much benefited by the administration of that medicine, when there is not any venereal taint even supposed to exist in the system; consequently, the ulcer produced in this case might have been induced to cicatrise during the exhibition of that mineral.

Till Mr. M. clears up the obscurities that involve his account of this case, I must still persevere in being of opinion, that the ophthalmia did not arise from any *venereal*, but from some *virulent* matter, applied to the eyes of the infant during its entré into the world.

I shall now beg leave to call Mr. M's attention to a paper published in the Edinburgh Medical and Surgical Journal for April, 1807, page 159, by Mr. Gibson of this town, a gentleman distinguished as a scientific surgeon, and deservedly eminent in his profession. Mr. G. gives it as his opinion, that the purulent ophthalmia is produced by the application of the matter of leucorrhœa to the eyes of the infant; and, after giving a case, mentions, that in the course of his practice, as surgeon to the Infirmary, he had seen during the last year, thirty-five cases, in all of which the mother had been affected with fluor albus during pregnancy. In the same work for April 1808, page 247, is an extract of a letter from Mr. Hill, of Chester, to the Editors, in which he relates a case of the purulent ophthalmia. He is rather of opinion, that it was induced by exposing the child to the heat of a fire immediately subsequent to its birth; but he acknowledges the mother was affected with leucorrhœa.

I have seen, this morning, at the Infirmary, three cases of the disease, patients of Mr. Gibson's; in all of which, the women were unwell with fluor albus, during utero-gestation; and, what decides the case in point, the complaint *existed before marriage*.

I would not wish Mr. M. to infer from any thing contained in this letter, that I am of opinion the matter of gonorrhœa, or venereal sores, *will not* induce a disease similar in appearances to the purulent ophthalmia of new-born children. I am fully aware that it *will*, and frequently *does*. I only contend, that the disease, *strictly so called*, is *induced by the application of the discharge from leucorrhœa*.

I am, &c.

PHILO.

*Manchester Infirmary, August* 1, 1808.

